# Variations in the Multilevel Structure, Gelatinization and Digestibility of Litchi Seed Starches from Different Varieties

**DOI:** 10.3390/foods11182821

**Published:** 2022-09-13

**Authors:** Xin Zhang, Lei Zhao, Wanxia Zhou, Xuwei Liu, Zhuoyan Hu, Kai Wang

**Affiliations:** 1 College of Food Science, South China Agricultural University, Guangzhou 510642, China; 2 Guangdong Laboratory for Lingnan Modern Agricultural, Guangzhou 510642, China; 3 Guangzhou Uniasia Cosmetics Technology Co., Ltd., Guangzhou 510640, China

**Keywords:** litchi seed, starch, structure, thermal properties, digestibility

## Abstract

Litchi seed starches from six varieties, as compared with maize starch, were studied for their multilevel structures, thermal and digestion properties to understand the distinct feather of each variety and provide guidance for their utilization in multi-industries. The results showed different varieties of litchi seed starch shared similar appearances with granules in oval shape and with a smooth surface. Starch granules of all the varieties exhibited typical bimodal size distributions consisting of small (<40 μm) and large granules (40–110 μm), although their relative proportions were largely dependent on variety. Huaizhi had the largest D50 value, whilst Guiwei showed the lowest. All the litchi seed starches had A-type crystalline with relative crystallinity varying from 20.67% (Huaizhi) to 26.76% (Guiwei). Similarly, the semi-crystalline structure varied apparently with variety. As to the chain-length distribution, only slight differences were observed among varieties, except Huaizhi displayed apparently higher amylose content (34.3%) and Guiwei showed the lowest (23.6%). Significant differences were also present in the gelatinization properties. Huaizhi seed starch showed significantly higher gelatinization temperatures and lower enthalpy change than the others. The digestibility of cooked litchi seed starches was only slightly different among varieties, suggesting variety is not the most critical factor regulating the digestibility of cooked litchi seed starch.

## 1. Introduction

Litchi (*Litchi chinensis* Sonn.), belonging to the Sapindaceae family, is widely distributed in tropical and subtropical areas of the world. Litchi fruit is one of the most favored subtropical fruits worldwide, due to its nutritional value and excellent taste [1,2]. Litchi seeds are the major by-product of litchi processing. They are normally discarded as waste in litchi fruit consumption and processing, leading to pressure on environment and resource waste. Litchi seeds have been used in Chinese traditional medicine to treat some diseases [1,3]. Studies in recent years found that litchi seeds are rich in bioactive compounds, including polysaccharides [4], polyphenols [5], proanthocyanidins [6], etc. Therefore, they are with multiple bioactivities, including anti-diabetic effects [5], inhibition of α-glucosidase activities [7], antioxidant and anti-tyrosinase activities [6,8], etc.

Litchi seeds are also an abundant source of starch, containing about 52.8% (dry weight of seeds) of starch [9]. Therefore, studies on starch from litchi seeds could be beneficial for its development and utilization in food and non-food industries.

Starch is a high molecular weight carbohydrate polymer comprised of condensed glucose subunits. It provides half of the energy for human activity; therefore, is an important ingredient for many foods. Starch has also been widely used in non-food industries, for example, as fillers, disintegrants and glidants because it is low-cost, easily available and non-toxic [10,11]. Typically, starch is supplied by traditional staple food materials, such as cereals, roots and legumes. Nevertheless, the starchy crops might be reduced due to urbanization that land for growing crops may be used for human activities [12]. It has been predicted that the price of staple grains will rise by 30–70% from 2000 to 2050 in the absence of climate change, and even over 100% in the presence of climate change [13]. This would lead to a dramatic increase in the cost of producing starchy foods from corps. Therefore, in recent years, increasing interest has been attracted to find unconventional starches from different plant resources, such as jackfruit, longan seeds, mango kernels, turmeric, and so on [11,14,15,16,17]. As an abundant source of starch, litchi seeds would have promising potential for providing starch for various industrial applications such as human food, animal feeds, paper making, pharmaceutical products, etc. To date, the basic physiochemical properties of litchi seed starch from a random variety have been studied [9,14,15]. However, the structural and physicochemical characteristics of litchi seed starch may vary largely with the variety, planting conditions and maturity stage of litchi, and this would have a dramatic impact on the application properties of litchi seed starch. Hence, understanding the structure and properties of litchi seed starch from different litchi varieties would be critical for its industrial application in the future.

In the present study, six litchi varieties distributed in South China were selected. Starch was extracted from litchi seeds, after which the multilevel structures of starch were characterized by scanning electron microscopy (SEM), laser particle size analyzer, small-angle X-ray scattering (SAXS), X-ray diffraction (XRD) and size exclusion chromatography (SEC), and the gelatinization and digestibility of starch were investigated using a differential scanning calorimeter (DSC) and an in vitro digestion model, respectively. These structural and property features of commercial maize starch were also studied for comparison. The results would provide detailed information for revealing the complex structure, physiochemical and nutritional properties of litchi seed starch from different varieties, which would give guidance for the utilization of litchi seed starch in various industries.

## 2. Materials and Methods

### 2.1. Materials

Litchi fruits from six litchi varieties, including Guiwei (GW), Jingganghongnuo (JGHN), Lizhihuang (LZH), Jizuili (JZL), Huaizhi (HZ) and Shuangjianyuhebao (SJYHB) were collected after full maturity from orchards in Guangdong, China. Maize starch (amylose content ~29%) was purchased from Qinhuangdao Lihua Starch Co., Ltd. (Qinhuangdao, China).

*α*-Amylase from porcine pancreas was purchased from Sigma-Aldrich Pty. Ltd. (St. Louis, MO, USA). Isoamylase (from *Pseudomonas* sp.) was purchased from Megazyme International, Ltd. (Bray Co., Wicklow, Ireland). Pullulan standards were purchased from Polymer Standards Service (PSS) GmbH (Mainz, Germany). Chemical reagents including sodium chloride, sodium metabisulfite, sodium carbonate and sodium hydroxide were used as received.

### 2.2. Starch Isolation

Isolation of starch from litchi seeds was carried out following the method described by Wang et al. [18]. Litchi seeds were crushed and soaked in sodium metabisulfite solution (0.45%, *w*/*v*) overnight. The mixture was then ground into a slurry using a homogenizer (IKA, Wilmington, NC, USA), and then filtered through a sieve with openings of 106 μm. The residues were homogenized again with sodium metabisulfite solution until the amount of residue on the sieve remained constant. The filtrate was combined, and then sodium chloride (NaCl, 0.1 M) and toluene (at a ratio of 9:1) were used to remove proteins and lipids. After constantly stirring the mixture for about 1.5 h, it was kept still until the upper toluene layer and NaCl layer could be easily removed. This step was repeated until the toluene layer became clear. The starch sample precipitated at the bottom was collected, and washed with water and ethanol prior to being dried at 40 °C.

### 2.3. Granule Morphology

Morphological characteristics of starch samples were observed using a scanning electron microscope (SEM, EVO18, Zeiss, Oberkochen, Germany). The dried starch samples were fixed on aluminum stubs with double-sided adhesive tape. Additionally, the mounted samples were coated with a film of platinum using a sputtering coater (Cressington Scientific Instruments, Watford, UK). Images were captured under an accelerating voltage of 10 kV at 4000× magnification [19].

### 2.4. Particle Size Distribution

The particle size distribution of starch samples was measured using a Mastersizer 3000 laser particle size analyzer (Malvern Instruments, Malvern, UK). The refractive index of starch granules and distilled water in the system were 1.54 and 1.33, respectively [20].

### 2.5. Crystalline Structure

The crystalline structure of starch samples was analyzed using an X-ray diffractometer (XRD, D8 Advance, Bruker, Bremen, Germany). The radiation source of the XRD was Cu-Kα, operating at 40 kV and 40 mA. The scanning speed of the instrument was set at 2°/min. A diffraction pattern with a scanning range of 4° (2θ) to 35° (2θ) was obtained, and the relative crystallinity was calculated by the ratio of the peak area of the crystal to the total diffraction area using PeakFit software (Systat Software Inc., San Jose, CA, USA) [19].

### 2.6. Semi-Crystalline Structure

The semi-crystalline of starch was analyzed using a small angle X-ray scattering (SAXS) instrument operated at 50 mA and 40 kV fitted with a pinhole collimation system. The X-ray source was a copper rotating anode, equipped with cross-coupled Göbel mirrors, and the Cu-Kα radiation wavelength was 1.54 Å. The samples were stored in a sealed cell to prevent dehydration and processed in X-ray monochromatic light for 1 min. Scatter detection was performed within the range of q value of 0.03–0.15 nm^−1^. A series of SAXS parameters, including peak intensity (I_max_), peak position (S_max_) and peak full width at half maximum (ΔS) were extracted from SAXS patterns using a graphical method [21]. The average thickness of semi-crystal (D), representing the lamellar distance, was calculated from S_max_ according to D = 2π/S_max_ [22].

### 2.7. Molecular Structure

The chain length distribution (CLD) of litchi seed samples was characterized using a size exclusion chromatography system (SEC, Agilent 1260 Infinity, Agilent, Santa Clara, CA, USA) with a refractive index detector (RID, Optilab UT-rEX, Wyatt, Santa Barbara, CA, USA) after debranching of starch using isoamylase. Starch solution (0.6 mL/min) was injected into the SEC system using DMSO containing 0.5% (*v*/*v*) LiBr as eluent, and molecules were separated using pre-, Gram 100 and Gram 1000 columns (Polymer Standards Service, Mainz, Germany) at 80 °C [18].

### 2.8. Thermo Properties

The thermal properties of starch samples were analyzed using a differential scanning calorimeter (DSC 8000, Perkin Elmer, Norwalk, CT, USA). The starch sample (3–4 mg, dry basis) was mixed with water (9–12 μg) and sealed in an aluminum pan [23]. After equilibrating at room temperature for 1 h, the sealed pan was heated from 30 °C to 95 °C at a rate of 10 °C/min. The enthalpy change (ΔH), onset (T_o_), peak (T_p_) and conclusion (T_c_) temperatures were calculated using Pyris software (Perkin Elmer, Norwalk, CT, USA).

### 2.9. In Vitro Digestion

Starch digestion was performed in vitro following a previously published method [18]. Each tube containing starch (50 mg, dry basis) and 10 mL of phosphate buffer solution (PBS, pH 7.4) was placed in a boiling water bath for 30 min with constant mixing to allow complete gelatinization of the starch. After keeping the mixture at 37 °C for 15 min, 1 mL of enzyme solution containing 2 units of α-amylase was added to hydrolyze the starch. After 0, 10, 20, 30, 60, 90, 120 and 180 min, a proportion of hydrolysate (0.3 mL) was taken and immediately mixed with sodium carbonate solution (0.5 M, 1.2 mL) to inactivate the enzyme. The mixture was centrifuged, and the supernatant was used to quantify the concentration of reducing sugars using para-hydroxybenzoic acid hydrazide (PAHBAH) method with maltose as the standard. Based on the absorbance value measured by the UV spectrophotometer (Uvmini-1240, Shimadzu, Tokyo, Japan) at λ = 410 nm, the reducing sugar was expressed as maltose equivalent, and the digestion curve was fitted to the first-order kinetics using the Log of Slope (LOS) plots.

ln(dC_t_/d_t_) = −kt + ln(C_∞_) (1)

where k is the digestion rate coefficient, C_t_ is the concentration of starch at digestion time t and C_∞_ is the corresponding concentration of starch at the end of digestion.

### 2.10. Statistical Analysis

The results were expressed as the average and standard deviation of at least duplicated measurements calculated by one-way analysis of variance (ANOVA), and Duncan’s multiple range tests were used to analyze data in SPSS statistical software (SPSS Inc., Chicago, IL, USA). Significant differences in the mean values were determined at *p* < 0.05.

## 3. Results

### 3.1. Morphological Characteristics

**Figure 1:**
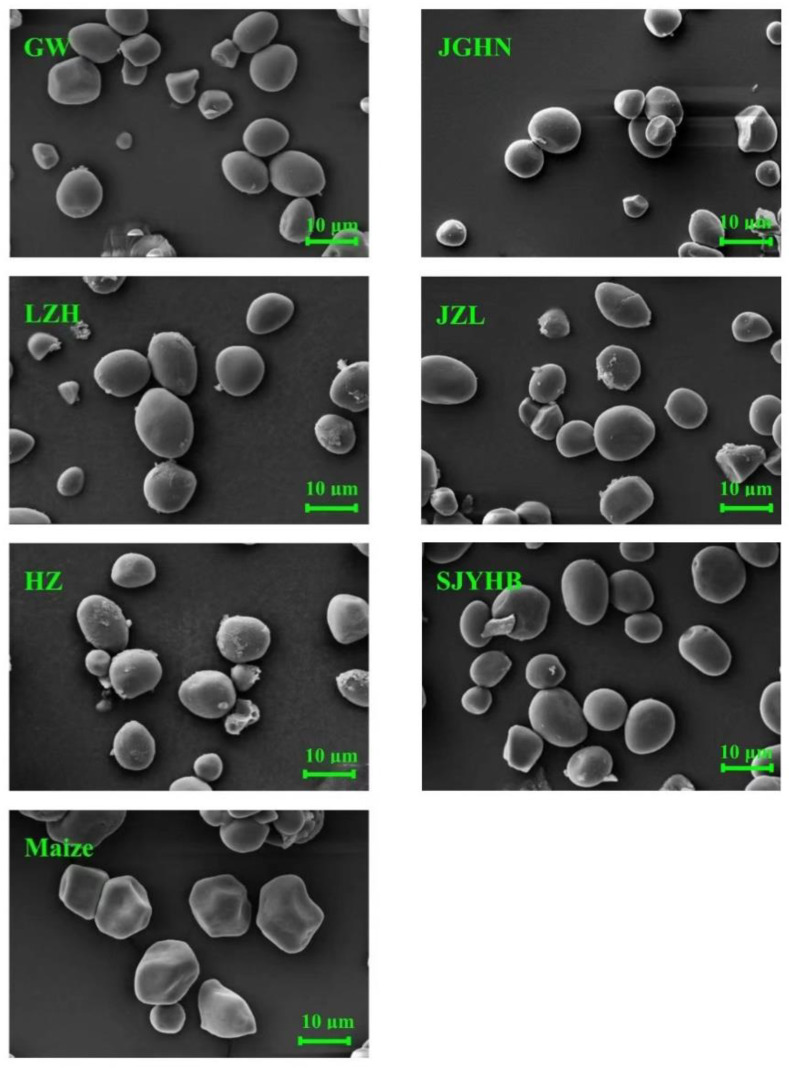
SEM images of litchi seed starches from different litchi varieties as compared with maize starch. GW, Guiwei; JGHN, Jingganghongnuo; JZL, Jizuili; LZH, Lizhihuang; HZ, Huaizhi; SJYHB, Shuangjianyuhebao.

The SEM images of litchi seed starches in comparison with maize starch are exhibited in Figure 1. Maize starch exhibited an oval and polygonal shape with sharp edges and pores on the surface, which is consistent with previous reports [24]. Differently, litchi seed starches were mainly with round to oval shapes with no pores or fissures on the surface, and a minority of granules were in polygonal shape. Starches from different varieties of litchi seeds were generally similar in their granule shape and surface morphology, indicating that each biological source has a set of common feature characteristics, and genetic diversity and growth environmental conditions had no significant effect on the granule morphology of litchi seed starch. In addition, there are some very small granules in all the litchi seed starches, although the majority are large granules, indicating the granular size may have bimodal distributions.

### 3.2. Particle Size Distribution

**Figure 2:**
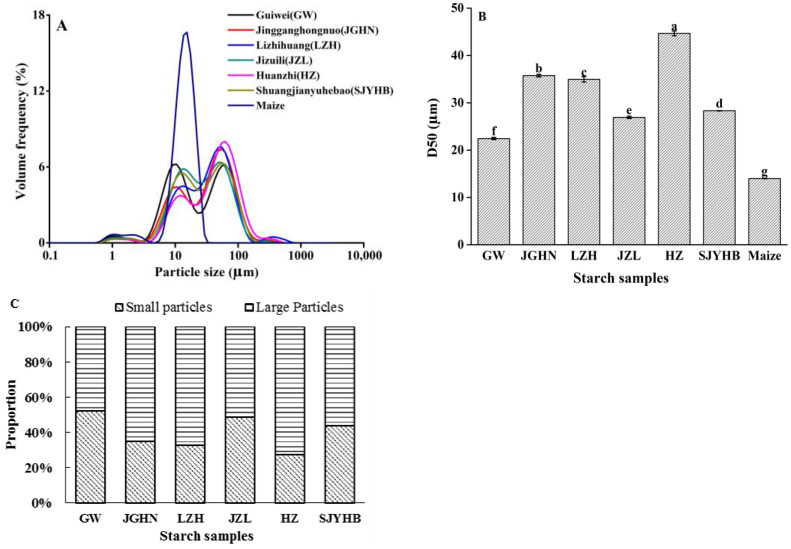
Particle size distributions (**A**), D50 values (**B**) and relative proportions of small and large particles (**C**) of litchi seed starches as compared with normal maize starch. Different letters in panel B indicate values are significantly different at *p* < 0.05.

The particle size distribution of starch samples was analyzed using a laser particle size analyzer, and the results are displayed in Figure 2. Normal maize starch displayed a unimodal size distribution, which is consistent with previously reported results [25]. Differently, all the litchi seed starches showed bimodal size distributions, suggesting there are two fractions containing small particles (ranging from 0.1 μm and 40 μm) and large particles (ranging from 40 to 110 μm) (Figure 2A).

The D50, representing the size at which 50% of the sample is smaller and 50% is larger [26], was also obtained (Figure 2B). The results showed that the D50 value of litchi seed starches were ranging from 22.46 (for Guiwei) to 44.67 (for Huaizhi) μm, which were significantly higher than maize starch (13.99 mm). In addition, there were also dramatic variations among different varieties of litchi seed starches. The relative proportions of the small and large particles of the litchi seed starches were obtained from the area under the distribution curve of each fraction, and the results are displayed in Figure 2C. Among the litchi varieties, Huaizhi seed starch had the highest proportion of large particles (72.69%), which contributes to its highest D50 value. Lizhihuang and Jingganghongnuo showed lower proportions of large particles (67.46 and 64.89%, respectively), followed by Shuangjianyuhebao (56.13%) and Jizuili (51.45%). Guiwei, on the other side, exhibited the lowest proportion of large particles (47.94%), which is consistent with its lowest D50 value. The differences in the particle size distribution of starch from different varieties of litchi seeds may lead to variations in their physiochemical and application properties. As previously reported, the particle size of starch granules is related to the physiochemical properties of rice starch [27], the baking properties of frozen dough [28], the quality of Chinese noodles, [29] etc.

### 3.3. Crystalline Structure

**Figure 3:**
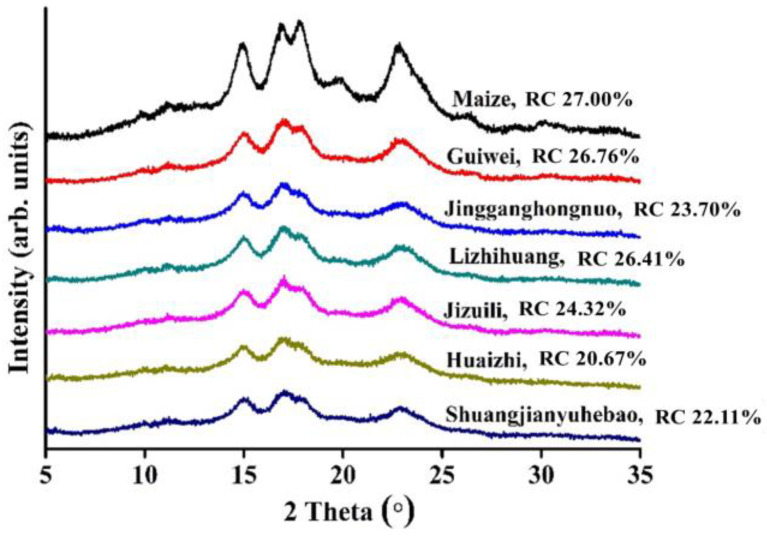
XRD patterns and relative crystallinity (RC) of litchi seed starches and maize starch.

The crystalline structure of starch was analyzed using XRD, and the resulting patterns and relative crystallinity of various litchi seed starches, in comparison with maize starch, are shown in Figure 3. Based on XRD patterns, starch from different sources normally can be classified into four types: A-, B-, C- and V-type [30]. As can be seen from Figure 3, all the litchi seed starch samples displayed similar XRD patterns to maize starch, with four main diffraction peaks at around 15°, 17°, 18° and 23° (2θ), respectively. These indicate A-type crystalline structure, which is usually reported in cereal starch [31]. Thory et al. [14] have previously reported A-type crystalline structure of a litchi seed starch from India. Differently, Guo, Lin, Fan, Zhang and Wei [9] reported CA-type (a mixture of A- and B-type, and similar to A-type) in an unknown variety of litchi seed starch from China. These inconsistencies might have resulted from the differences in the variety and environmental conditions, which have been reported as having important influences on starch structure [31].

In terms of the crystallinity, apparent differences were present among litchi seed starches from different varieties, varying from 20.67% to 26.76%. Guiwei and Lizhihuang showed the highest crystallinity values (26.76% and 26.41%), followed by Jizuili (24.32%) and Jingganghongnuo (23.70%). In contrast, Huaizhi and Shuangjianyuhebao exhibited the lowest crystallinity values (20.67% and 22.11%, respectively). These suggest there are apparent differences in the crystalline structure of litchi seed starches from different varieties.

### 3.4. Semi-Crystalline Structure

**Figure 4:**
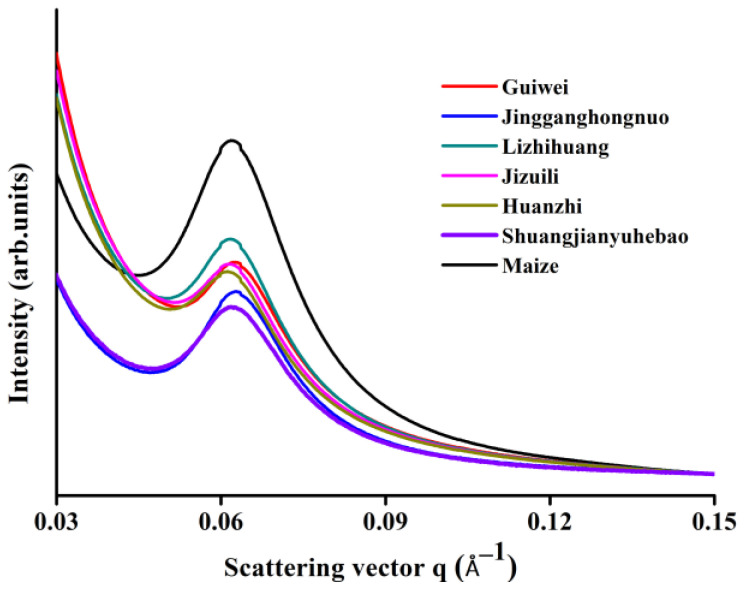
SAXS spectra of litchi seed starches and maize starch.

The semi-crystalline structure, composed of alternating amorphous and crystalline regions, was characterized using SAXS with results exhibited in Figure 4. To eliminate variations in sample concentration, results were adjusted to equal intensity at q = 0.15 nm^−1^ to allow direct comparisons. Parameters including peak intensity (I_max_), peak position (S_max_), peak full width at half maximum (ΔS) and average thickness of semi-crystal (D) were obtained from SAXS spectrum and summarized in Table 1.

The SAXS patterns of all starch samples showed obvious scattering peaks at about q = 0.06 nm^−1^, and the peak position S_max_ values are as shown in Table 1. Theoretically, the position of the peak (S_max_) is inversely proportional to the average total thickness (i.e., lamellar repeat distance or Bragg spacing) of the periodic arrangement lamellar architecture [32], which is expressed as D values and exhibited in Table 1. The results showed that the D values of litchi seed starches and maize starch were close (ranging from 9.96 to 10.27 nm), which are in agreement with the lamellar thickness of other previously reported starches (9–10 nm) [33,34,35]. This suggests that the lamellar repeat distance is constant and independent of starch fine structure including the starch crystalline type, crystallinity, particle size, etc.

The ΔS values of litchi seed starches from different varieties were ranging from 0.013 to 0.017, which is slightly lower than that of maize starch (0.019). The I_max_ value of maize starch (174.25) was 1.2 to 2.6 times of that of litchi seed starches, among which Huaizhi had the lowest I_max_ value (68.28) and Lizhihuang had the highest (144.78). As previously reported, I_max_ is related to the amount of ordered semi-crystalline structures and the differences in electron density between crystalline and amorphous lamellae [21]. These results suggest that there are apparent differences in the semi-crystalline structure of litchi seed starches among varieties.

### 3.5. Molecular Structure

**Figure 5:**
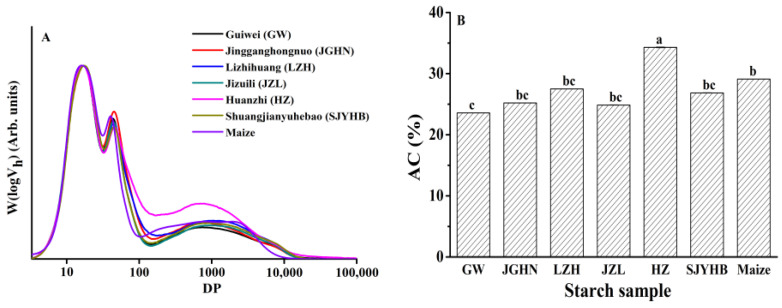
Chain length distribution (CLD) (**A**) and amylose content (AC) (**B**) of litchi seed starches as compared with maize starch. Different letters in panel B indicate values are significantly different at *p* < 0.05.

The chain length distribution of debranched litchi seed starches and maize starch, after being normalized to the same global maximum to allow comparison among samples, are exhibited in Figure 5A.

Typical chain length distribution profiles were observed for all the starch samples, with two amylopectin peaks at around DP 17 and 47, respectively, and a broad amylose peak at approximately DP > 100. This is similar to previously published results of starch from various botanical sources [18]. And as previously reported, the first amylopectin peak at DP 17 represents short amylopectin chains (A and B1 chains) staying within one crystalline/amorphous layer, and the second peak at about DP 47 represents longer amylopectin chains (B2 and longer chains) spanning at least two crystalline layers. The broad peak at DP ≥ 100 contributes to mainly amylose chains [36]. Taking a closer look at these chain length distributions, it was apparently shown that there are differences among different varieties of litchi in the height of long amylopectin peak (at around DP 47) and amylose peak (at DP > 100), which suggests differences in the proportion of each category of chains. For example, Huaizhi showed higher peaks of amylose (at DP > 100) and a lower peak of long amylopectin chains at DP about 47, implying it has a higher proportion of amylose, and a lower proportion of long amylopectin chains compared with other varieties. On the other hand, Jingganghongnuo exhibited a higher peak at DP 47 compared with other litchi varieties, which suggests its higher proportion of long chain amylopectin.

The amylose content was also calculated based on the ratio of area under amylose curve (DP < 100) to that under the whole distribution curve. As summarized in Figure 5B, maize starch showed an amylose content of 29.1%, which is similar to previously reported results [18]. Litchi seed starch showed variations in the amylose content among different varieties, with Huaizhi exhibiting the highest value (34.3%) and Guiwei showing the lowest (23.6%). Statistically similar amylose content results were observed for the other four varieties, ranging from 24.86% to 27.50%. These results suggest that the molecular structure of starch in litchi seeds largely depends on the varieties of litchi, which is similar to starch from other botanical origins [18].

### 3.6. Thermal Properties

The thermal properties, including the gelatinization temperatures (onset temperature T_o_, Peak temperature T_p_ and conclusion temperature T_c_) and the enthalpy change (ΔH), of litchi seed starches and maize starch were analyzed using DSC, with results being summarized in Table 2.

It was observed that for maize starch, the temperature range of the gelatinization process of starch samples was between 64.03 °C (T_o_) and 75.60 °C (T_c_), and the ΔH was 6.12 J/g. In comparison, all the litchi seed starches showed higher gelatinization temperatures (including T_o_, T_p_ and T_c_) and lower ΔH. Among the litchi varieties, Shuangjianyuhebao showed the lowest onset (68.86 °C) and peak (75.87 °C) temperatures, whilst Huaizhi showed the highest (75.04 and 80.04 °C, respectively). The other four litchi varieties showed similar gelatinization temperatures, with no significant differences. A different trend was observed in the ΔH of litchi seed starches, with Guiwei displaying a significantly higher ΔH value (9.41 J/g) than the other varieties. The gelatinization temperatures reflect the heat stability of crystallites, while ΔH reflects the energy needed to disrupt the molecular order of the starch [36]. The current results demonstrated significant differences in the gelatinization properties of litchi seed starches from different varieties, which is probably related to variations in the crystalline and -semi-crystalline structure of these starches.

### 3.7. In Vitro Digestion

**Figure 6:**
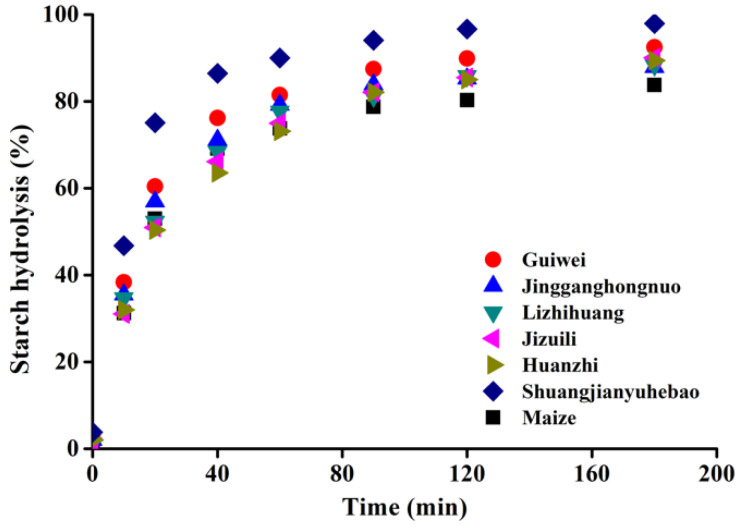
Starch digestion patterns of completely gelatinized litchi seed starches and maize starch.

The digestion properties of different varieties of litchi seed starches and maize starch were analyzed in vitro using α-amylase after complete gelatinization of the starch in water to simulate the intestinal digestion process of cooked starch in foods. The digestion curves are displayed as the proportion of digested starch as a function of digestion time in Figure 6. Similar digestion profiles are shown for all the starch samples, with the proportion of hydrolyzed starch being progressively increased with a prolonged digestion time. After initiation of digestion, starch was relatively quickly hydrolyzed in the first 40 min, after which the digestion progress slowed down progressively, reaching a plateau after approximately 180 min.

In order to make quantitative comparisons among samples, the digestion curves were fitted with first-order kinetics, and the resulting digestion rate coefficient (k) are exhibited in Table 2. The results showed there are only slight differences in the digestion rate coefficient among litchi varieties. Huaizhi seed starch had a slightly lower k value (0.030 min^−1^) than others, while Shuangjianyuhebao showed the highest digestion rate (0.044 min^−1^). Maize starch had a k value of 0.041 min^−1^, which is not statistically different from most of the litchi seed starches. These suggest that the digestion rate of gelatinized starch from different varieties of litchi seeds are generally similar to each other, and it is not related to the structural differences among varieties.

This is in accordance with previously published results reporting no dramatic differences in the digestibility of starch from different botanical sources [18]. The reason is probably because cooking starch in sufficient water allows complete gelatinization, leading to the destruction of the ordered structure and granular structure of starch molecules; therefore, the ordered structure and granular structure have no impact on the digestibility of starch. This is different from starch in the food matrix, where starch digestibility may be influenced by the cell integrity, cell wall structure, starch gelatinization degree and other food components such as protein and lipid [18].

## 4. Conclusions

In this study, starch was isolated from litchi seeds of six varieties. The structural characteristics of these starches were analyzed, and gelatinization and digestibility were studied. The results showed litchi seed starches were mainly in round to oval shape with smooth surfaces, and no apparent differences were observed among varieties, implying these are common features for litchi seed starch. Typical bimodal granule size distribution patterns were exhibited for all the varieties, indicating litchi seed starches consist of both small and large granules with size <40 μm and 40–110 μm, respectively. The D50 and relative proportions of the small and large particles were dramatically different among varieties: Huaizhi had more large granules (72.69%), while Guiwei had the lowest (94%). All the starches were with A-type crystalline structure, whereas the relative crystallinity apparently vary with variety. Guiwei had the highest crystallinity (26.76%) and Huaizhi had the lowest (20.67%). The semi-crystalline structure was also different among varieties. In terms of the molecular structure, only slight differences were present in the CLDs among varieties, with Huaizhi exhibiting apparently different CLD pattern and significantly higher amylose content (34.3%).

Regarding thermal properties, Huaizhi showed significantly higher gelatinization temperatures and lower ΔH than the others, while Guiwei had the highest ΔH. The digestion properties of gelatinized starch samples suggest only slight differences among varieties, and it does not seem associated with variety. These results would be beneficial for better understanding the characteristics of litchi seed starches, and provide guidance for their utilization as a novel source of starch in multiple industries.

## Figures and Tables

**Table 1:** SAXS parameters of litchi seed starches and maize starch.

Samples	Imax (Counts)	ΔS (Å^−1^)	Smax (Å^−1^)	D (nm)
Guiwei	77.61	0.013	0.063	10.03
Jingganghongnuo	100.00	0.015	0.063	9.96
Lizhihuang	144.78	0.014	0.062	10.17
Jizuili	73.88	0.013	0.062	10.16
Huaizhi	68.28	0.014	0.061	10.27
Shuangjianyuhebao	83.21	0.017	0.062	10.17
Maize	174.25	0.019	0.062	10.19

**Table 2:** Thermal properties and digestion rate coefficient of litchi seed starches and maize starch *.

Samples	T_o_ (°C)	T_p_ (°C)	T_c_ (°C)	ΔH (J/g)	k (min^−1^)
Guiwei	74.27 ± 0.11 ^b^	78.40 ± 0.00 ^b^	83.24 ± 0.13 ^c^	9.41 ± 0.37 ^b^	0.039 ± 0.009 ^ab^
Jingganghongnuo	73.97 ± 0.04 ^b^	78.22 ± 0.01 ^b^	82.80 ± 0.00 ^c^	6.48 ± 0.03 ^c^	0.040 ± 0.000 ^ab^
Lizhihuang	73.85 ± 0.28 ^b^	78.32 ± 0.11 ^b^	82.96 ± 0.10 ^c^	7.08 ± 0.06 ^c^	0.034 ± 0.003 ^ab^
Jizuili	73.71 ± 0.25 ^b^	78.23 ± 0.01 ^b^	83.00 ± 0.18 ^c^	7.62 ± 0.60 ^c^	0.033 ± 0.006 ^ab^
Huaizhi	75.04 ± 0.25 ^a^	80.04 ± 0.22 ^a^	84.64 ± 0.24 ^a^	6.12 ± 0.86 ^c^	0.030 ± 0.004 ^b^
Shuangjianyuhebao	68.86 ± 0.3 ^c^	75.87 ± 0.48 ^c^	84.08 ± 0.46 ^b^	7.30 ± 0.09 ^c^	0.044 ± 0.004 ^a^
Maize	64.03 ± 0.07 ^d^	70.06 ± 0.25 ^d^	75.60 ± 0.17 ^d^	13.43 ± 0.33 ^a^	0.041 ± 0.007 ^ab^

* Different letters in the same column indicate values are significantly different at *p* < 0.05. T_o_, T_p_ and T_c_ refer to onset, peak and conclusion temperatures, respectively. ΔH is the enthalpy change, and k is the digestion rate coefficient.

## Data Availability

Data is contained within the article.
